# Public preferences for primary care provision in Germany – a discrete choice experiment

**DOI:** 10.1186/s12875-019-0967-y

**Published:** 2019-06-11

**Authors:** Kim-Sarah Krinke, Ulla Tangermann, Volker Eric Amelung, Christian Krauth

**Affiliations:** Hannover Medical School, Institute for Epidemiology, Social Medicine and Health Systems Research, Carl-Neuberg-Straße 1, 30625 Hannover, Germany

**Keywords:** Primary care, Preferences, Discrete choice experiment

## Abstract

**Background:**

Primary care is a central element of healthcare and addresses the main health problems of the population. While primary care gains in importance due to an aging population, there is an ongoing debate on physician shortages in German rural regions. The study aims on analyzing the population’s preferences on primary healthcare and, therefore, on helping policy makers to make care delivery more responsive to patients’ needs when planning political reforms of primary care.

**Methods:**

A paper-based discrete choice experiment (DCE) was used to assess preferences of the population of eight rural regions in Germany. Based on literature search and qualitative research, six attributes were selected and included in the choice experiment. The survey presented participants with eight choice sets in which they had to choose between two possible scenarios of care. A conditional logistic regression as well as a latent class model (LCM) were used to analyze preferences for primary healthcare.

**Results:**

Nine hundred four participants completed the survey (response rate 46.1%). The conditional logistic regression showed significant impact of the attributes “home visits”, “distance to practice”, “number of healthcare providers”, “opening hours of the practice”, and “diagnostic facilities” on the respondents’ choices of primary healthcare alternatives. Moreover, the LCM identified four classes that can be characterized by preference homogeneity within and heterogeneity between the classes.

**Conclusion:**

Although the study revealed heterogeneous preferences among the latent classes, several similarities in preferences for primary care could be detected. The knowledge on these public preferences may help policy makers when implementing new models of primary care and, thus, raise the populations’ acceptance of future primary care provision and innovative care models.

**Electronic supplementary material:**

The online version of this article (10.1186/s12875-019-0967-y) contains supplementary material, which is available to authorized users.

## Background

As key element of healthcare, primary care represents an essential part of a country’s health system and, thus, is highly important to most citizens [1]. Primary healthcare, which includes curative and preventive services as well as patient education on the major health problems, is healthcare that traditionally focuses on the needs of the patients, in the first place [2]. In an international comparison, the current German ambulatory care is characterized by a high density of physicians and a good access to care [3]. However, there is an ongoing public debate on shortages of physicians, especially in rural and remote areas in Germany. This discussion is predominantly based on two factors: the demographic change and the unequal regional distribution of outpatient physicians. There is a decreasing number of general practitioners and, simultaneously, an increasing number of specialists [4]. German health policy is aware of these processes and focuses on this issue, too. In recent years, there were several laws that aimed at improving the outpatient healthcare, for example the so called ‘Versorgungsstärkungsgesetz’ in 2015. This law intended to encourage the implementation of new and innovative models of care which supplement traditional forms of care provision. The latter ones are currently primarily based on physicians’ care. Such new models of care could include delegation, concepts based on mobility, like patient busses or mobile practices, or telemedical care. In other countries, such models of care have already been implemented, e.g. nurse practitioners in the UK and the US or telemedicine in sparsely populated areas of Finland. Those are adequate strategies to guarantee a needs-based medical supply despite a shortage of general practitioners (GPs) [5].

In Germany the GP often serves as the first contact person in cases of health complaints and patients have confidence in their GP due to long-term relationships [6]. A restructuring of primary care may, thus, only be successful if it is accepted by the general public or the patients, respectively. Otherwise patients would probably not make use of these new models of primary care. There are various aspects of primary care, which are important to the population [2]. With regard to the limited financial and personal resources it is virtually impossible to meet all patients’ expectations concerning primary healthcare provision. Thus, policy makers should be aware of aspects of primary care that are considered as particularly important by the population and of the trade-offs they are willing to make between various aspects of care. This knowledge should be taken into account when planning political reforms of primary care [7].

This study aims at assessing the population’s preferences for primary healthcare provision in Germany. To that end, a discrete choice experiment (DCE) was conducted to assess preferences for a range of characteristics of primary care provision in different rural areas in Germany.

## Methods

The DCE is a stated-preference technique that is often used in health economics research to elicit preferences for health programs or products [8]. It is an attribute-based-method of preference measurement in which respondents are asked to make hypothetical but realistic choices between two or more options of health products or services presented in a choice task. DCEs are based on Lancaster’s [9] theory according to which the utility of a good or service is determined by its different characteristics, called attributes. Each attribute has several levels that describe the range over which an attribute varies. By choosing between several choice alternatives the respondents value attributes against each other. Thus, preferences are revealed through the respondents’ choices [10, 11]. Typically, a DCE contains multiple choice tasks which are described by varying attribute levels. Through the use of a DCE, the relative importance of the attributes as well as the trade-offs between the attributes can be analyzed.

### Selection of attributes and attribute levels

The first step in a DCE is to select attributes and levels that adequately describe the good or service of interest. In our study the service of interest was primary healthcare provision. For identifying the relevant attributes and levels we firstly conducted systematic review of the existing literature where we identified a list of potential attributes and levels [12]. All attributes which were irrelevant for primary care in Germany, e.g. treatment costs and which were irrelevant for our research question were excluded from the list. As a second step, we conducted qualitative research in terms of focus group discussions (3 discussions with a total number of 17 persons). Each focus group comprised between four and seven participants of the adult population with diverse characteristics with regard to age, gender and health status. Based on an interview guideline, the participants discussed various aspects and attributes of primary care. The final selection of attributes and levels was driven by the intention to derive useful and practical recommendations from the study outcomes, especially with regard to innovative models of care. After a pilot study with 19 participants, who were not included in the main sample, we finally identified six attributes with the corresponding levels which are shown in Table 1.

**Table 1:** Attributes and attribute levels included in the DCE

Attributes	Levels
1. Home visits	• Home visits are provided • Home visits are not provided
2. Distance to practice	• 15 min • 30 min • 45 min
3. No. of healthcare providers	• Healthcare provision by one GP • Healthcare provision by varying GPs
4. Opening hours of the practice	• Practice opens 5 days a week • Practice opens 4 days a week • Practice opens 3 days a week
5. Delegation of medical tasks	• No delegation of medical tasks • Delegation of medical tasks to specially skilled professionals
6. Diagnostic facilities	• Extensive diagnostic facilities • Limited diagnostic facilities

### Experimental design

The experimental design of a DCE refers to how the attributes and the corresponding levels are combined into choice alternatives and choice sets [13]. On the basis of 3^2^*2^4^ = 144 possible combinations of attribute levels in a full factorial design, we conducted a fractional factorial design with 16 choice sets with 2 alternatives.^1^ The 16 choice sets were split up into 2 blocks with 8 choice sets each, that were incorporated into two versions of questionnaires. Thus, each respondent had to make 8 instead of 16 choices. We used SAS software (SAS Institute, Cary, NC, USA), which allows for the optimization of design efficiency, level balance, and the number of choice tasks. This design allows for a main effects model to be estimated, interactions between the attributes cannot be taken into account. To lower the cognitive burden for the respondents the 16 choice sets were randomly blocked into two questionnaire versions, each version containing eight generic choice tasks. Additionally, we included a fixed choice set offering two choice alternatives with one intended to be strictly dominant over the other to test for internal validity.

We did not include an opt-out or status quo option in the DCE because in the focus group discussions the participants clearly demonstrated that they are not willing to accept any kind of healthcare provision which is, in their perspective, worse than the current one. To avoid larger numbers of respondents who choose the opt-out option to prevent making challenging choices we decided to use binary choices and, thus, force a choice.

### Sampling and data collection

**Fig. 1:**
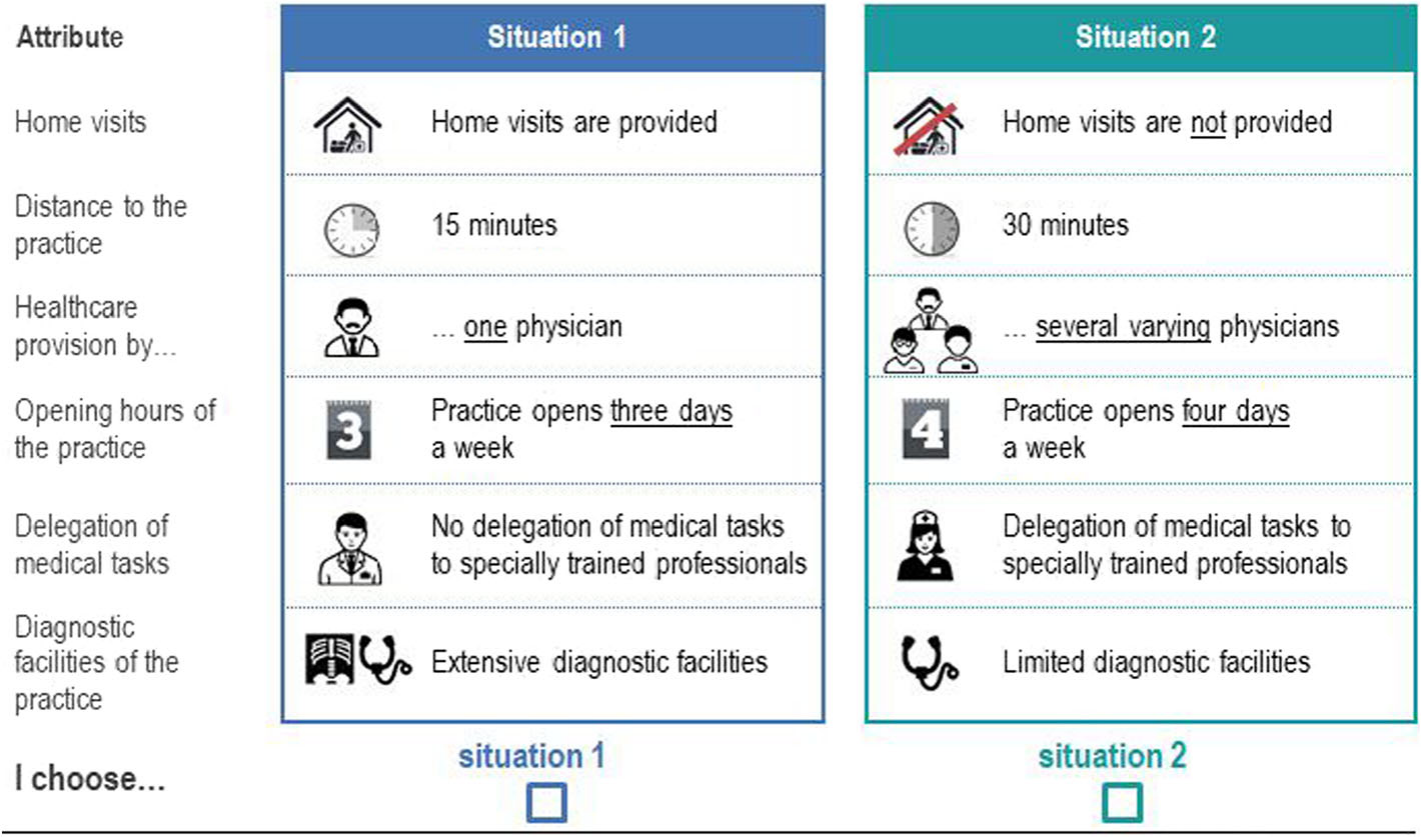
Example of a choice set

We collected the data through a self-complete postal questionnaire which was developed for this study (see Additional file 1 for an English version of the questionnaire). Altogether, the questionnaire contained three parts: Part one included questions concerning the present primary care provision. Part two consisted of the DCE and two questions for assessing the difficulty of the DCE and certainty when answering the choice tasks. The last part included socio-demographic questions. Figure 1 shows an example of a choice set which we illustrated with colors and symbols to keep the cognitive burden for the respondents as low as possible. The survey was conducted in summer of 2015 in eight rural areas in the federal state of Lower Saxony in Germany. The selection of the regions is described elsewhere [14]. After approval from the Ethics Committee of Hannover Medical School, we sent 2000 questionnaires to a random sample of the population aged 18 years and older drawn by the Residents’ Registration Offices. Information concerning the survey was provided in an enclosed personalized letter. The questionnaires were returned by the respondents via prepaid envelops. A reminder was sent two weeks after the shipping of the questionnaires.

Sample size was calculated based on a rule of thumb proposed by Johnson and Orme [15] and Orme [16]. Considering the number of choice tasks per person, the number of attributes and attribute levels, a sample size of *n* = 200 persons was needed. As we wanted to perform a subgroup analysis between hard to serve and normal to serve rural areas,^2^ we aimed at a total sample size of *n* = 400. Since self-complete postal DCE surveys often result in low response rates [10] we expected a response rate of about 20%.

### Data analysis

The collected data was imported into Stata version 14 (StataCorp LP, College Station, USA). The data analysis is guided by random utility theory (RUT) which assumes that individuals’ choices are affected by latent variables and that the utility of a good or service can be decomposed into an explainable, systematic and an unexplainable component [10, 17]. Utility can be obtained from a good’s attributes rather than from the good itself [9] and we consider the population to always choose the alternative with the highest utility.

The data from the DCE was analyzed using conditional logit regression, which is a commonly used method for examining DCEs [11]. However, as the conditional logit method has rather restrictive model assumptions, e.g. that it cannot take the DCE’s panel structure into account and does not account for preference heterogeneity, we also used a latent class model (LCM). LCMs assume that there are respondents with similar choices and preferences who can be grouped into latent classes. While preferences within a class are assumed to be homogeneous they differ between the classes [18]. The choice of the number of latent classes is exploratory, thus not initially determined, and is usually based on goodness-of-fit measures, such as log-likelihood ratio or information criteria. The calculation of relative importance of the attributes or attribute levels respectively allows for a comparison of preferences between the classes. Significant independent variables in the choice model point out that the attribute or level has a significant impact on the preferences for primary healthcare.

Additionally, we calculated marginal rates of substitution (MRS), so called trade-offs between two of the included attributes. The MRS can be calculated by partially differentiating the indirect utility function regarding attributes i and j (from the included attributes) and calculating their ratio:

$$ {MRS}_{X_{i,}{X}_j}=\frac{\raisebox{1ex}{$\partial V$}\!\left/ \!\raisebox{-1ex}{${\partial X}_i$}\right.}{\raisebox{1ex}{$\partial V$}\!\left/ \!\raisebox{-1ex}{${\partial X}_j$}\right.} $$

where *V* is an indirect utility function, *X*_*i*_ and *X*_*j*_ are two of the included attributes, and *∂* is the partial derivative [10, 19]. In this study, the MRS for the first two attributes, for example, can be interpreted as the respondents’ willingness to take into account an additional distances to access primary care provision, as the metric attribute “distance to practice” is the denominator of this equation.

## Results

The overall response rate was 46.1% (*n* = 904). The respondents were distributed equally across the two versions of the questionnaire, which only differed regarding the choice sets, with *n* = 449 respondents completing questionnaire version 1 and *n* = 455 completing version 2. There were six respondents (0.7%) who failed the internal validity check of the dominant choice presented in the DCE. Models estimated with and without these respondents did not result in any significant difference in the models and therefore, following current practice, these respondents were retained in the analysis [20, 21].

As shown in Table 2, respondents had a mean age of 56.6 years (SD 17.7). 53.3% were female and almost half of the respondents lived in a two person household. Moreover, 35.6% of the participants were working full time and 38.9% were retired. 53.7% rated their health status as good, 23.2% as less good and only 4.5% as poor. Overall 36.3% of the respondents had one or more chronic diseases.

**Table 2:** Characteristics of respondents

Characteristic	%
Age (mean) in years (SD)	56.6 (17.7)
Female	53.3%
Household size	
1 person	14.1%
2 persons	48.7%
3 or more persons	37.2%
Children under 18 years old	21.7%
Labor situation	
Working full time	35.6%
Working part time	11.7%
Retired	38.9%
Unemployed	7.3%
Other	6.4%
Health status	
Excellent	4.4%
Very good	14.2%
Good	53.7%
Less good	23.2%
Poor	4.5%
Chronic disease(s)	36.3%
Statutory health insurance	88.6%

### Conditional logit model

Table 3 presents the results of the conditional logit model. As the majority of the respondents (63.7%) found the choice tasks easy or rather easy to complete and 75.6% were rather sure or sure when making choices, the data validity can be considered high [22]. Except for the coefficient for delegation of medical tasks, all attributes or levels respectively were significant, indicating that they were relevant to the patient’s decision for a primary healthcare alternative. Moreover, the significant coefficients had the expected signs. The alternative specific constant was not significant, showing that respondents made their choice only on the basis of the attributes in the list. Positive values for the coefficients indicate that choice alternatives including this level are preferred over others. For the attribute “opening hours of the practice”, the level 3 days was determined as reference category.

**Table 3:** Conditional logit estimates of preferences for primary healthcare

	Coefficient	Standard error	*p*-value
Home visits are provided	0.938	0.036	0.000
Distance to practice (metr.)	−0.042	0.002	0.000
Healthcare provision through one GP	0.527	0.041	0.000
Practice opens 4 days a week (ref: 3 days)	0.753	0.062	0.000
Practice opens 5 days a week (ref: 3 days)	1.205	0.054	0.000
Delegation of medical tasks	0.061	0.043	0.155
Extensive diagnostic facilities	0.532	0.033	0.000
Constant	−0.010	0.060	0.866

*Metr.* metric, *GP* General practitioner, *ref.* reference category

Opening hours of 5 days a week (versus the reference category of 3 days) was the most important attribute level for the respondents, followed by the provision of home visits, opening hours of 4 days a week, extensive diagnostic facilities and the healthcare provision by one GP. The negative coefficient of the attribute “distance to practice” suggests that the respondents preferred a primary healthcare situation in which the practice is near their home.

Estimates based on *n* = 13,548 observations; Log likelihood = − 3353.0609; Bayesian Information Criterion (BIC) = 6782.234; Akaike Information Criterion (AIC) = 6722.122.

Based on the results of the conditional logit model marginal rates of substitutions or trade-offs between the attributes were calculated. Table 4 shows that the respondents are willing to drive further distances to the practice if it provides home visits (23 min), if a practice has opening hours of 4 days (18 min), or five days (29 min), or if a practice has extensive diagnostic facilities (13 min). Also, respondents are willing to accept a time distance of 13 min if healthcare is provided through a consistent GP rather than varying GPs.

**Table 4:** Marginal rates of substitutions (in minutes)

Attribute/Level	Coefficient	*p*-value
Home visits are provided	22.543	0.000
Healthcare provision through one GP	12.692	0.000
Practice opens 4 days a week	18.175	0.000
Practice opens 5 days a week	29.035	0.000
Delegation of medical tasks	1.371	0.261
Extensive diagnostic facilities	12.785	0.000

### Latent class model

The latent class estimates are presented in Table 5. As recommended in the literature, latent class estimates with different numbers of classes were compared with regard to the log-likelihood and information criteria, and also in terms of appropriate interpretation of classes [11, 23].

**Table 5:** Estimation of the latent class model

	Class 1		Class 2		Class 3		Class 4	
Class share	0.354		0.149		0.300		0.197	
	Coeff. (SE)	95% CI	Coeff. (SE)	95% CI	Coeff. (SE)	95% CI	Coeff. (SE)	95% CI
Home visits are provided (ref. no home visits provided)	1.311*** (0.213)	0.892–1.729	3.243*** (0.535)	2.194–4.291	0.604*** (0.119)	0.371–0.837	0.573*** (0.120)	0,337–0.810
Healthcare provision through one GP (ref: through changing GPs)	−0.108 (0.199)	− 0.498 – 0.282	− 0.011 (0.202)	− 0.502 –0.290	0.290* (0.131)	0.033–0.547	2.097*** (0.204)	1.697–2.497
Practice opens four days a week (ref: 3 days)	1.957** (0.646)	0.690–3.224	− 0.390 (0.351)	−1.078 –0.299	1.714*** (0.226)	1.271–2.157	−0.095 (0.212)	− 0.511 – 0.321
Practice opens five days a week (ref: 3 Tage)	2.363*** (0.644)	1.101–3.626	0.131 (0.219)	−0.299 – 0.562	2.784*** (0.326)	2.146–3.422	0.272 (0.127)	0.024–0.520
Delegation of medical tasks (ref: no delegation)	0.205 (0.184)	−0.156 – 0.565	1.147* (0.532)	0.104–2.190	0.030 (0.113)	− 0.192 – 0.252	−0.545*** (0.131)	− 0.803 – − 0.288
Extensive diagnostic facilities (ref: limited diagnostic facilities)	1.368*** (0.289)	0.802–1.933	−0.140 (0.442)	−1.007 – 0.726	0.463** (0.142)	0.186–0.742	0.918*** (0.39)	0.645–1.190
Distance to practice (metr., in minutes)	− 0.106*** (0.024)	−0.152 – − 0.060	−0.044* (0.022)	− 0.088 – 0.001	−0.048*** (0.007)	− 0.062 – − 0.035	−0.022** (0.007)	− 0.035 – − 0.008
No. of observations	13,562							
No. of persons	904							
Log-Likelihood	− 3038.661							
BIC	6077.322							
AIC	6077.322							
CAIC	5930.112							

*Metr.* metric, *GP* General practitioner, *ref.* reference category, *CI* confidence interval, *Coef.* coefficient, *SE* standard error, *ref.* reference category, *No.* number, *BIC* Bayesian Information Criterion, *AIC* Akaike Information Criterion, *CAIC* ‘consistent’ AIC; Significance: *p < .05, **p < .01, ***p < .001

Four latent classes, numbered from one to four, were identified. The respective class shares or memberships and coefficients as well as the standard errors and 95% confidence intervals are presented in the Table 5.

The class shares were 0.354 for class 1, 0.149 for class 2, 0.300 for class 3, and 0.197 for class 4. For the first class the coefficients for the attributes “home visits”, “opening hours” and “diagnostic facilities” had a significant positive effect and “distance to practice” a significantly negative effect on the class’s preferences for primary care provision. The attributes “number of healthcare providers” and “delegation of medical tasks” did not have significant effect on the preferences. In the second class, which is the smallest one, the provision of home visits had a strongly positive effect. Furthermore, there was a significantly positive effect for the delegation of medical tasks to especially skilled professionals. The distance to practice has a significant negative effect and the other attributes do not have significant effects on the class members’ preferences and therefore do not influence their choice of a primary care alternative. Class 3 has strong preferences for home visits, healthcare provision through only one GP and opening hour of 4 days or 5 days versus 3 days (reference category). Furthermore, they prefer extensive practice facilities and short distances to the practice. Compared to class 1, the coefficients for opening hours are considerably larger than for the other attributes so that members of class 3 had stronger preferences for this attribute. The fourth class showed significant preferences for the healthcare provision through one GP, large diagnostic facilities and the provision of home visits. The attributes “distance to practice” and “delegation of medical tasks” had negative effects and therefore negatively influenced the respondent’s choice for a healthcare situation presented in a choice set in this class.

## Discussion

All in all, the preferences for primary healthcare are significantly influenced by the provision of home visits, the distance to the practice, the healthcare provision by one or varying GPs, the practice’s opening hours, as well as the practice’s diagnostic facilities of a practice. The results of the conditional logit model show that especially the attribute “opening hours” strongly influenced the respondents’ choice decisions and is of relevance for primary healthcare. Furthermore, the provision of home visits is of high importance for rural population. The only attribute that has no significant influence on the participants’ preferences for primary care provision is the delegation of medical tasks. However, according to the results of the latent class analysis, there are some differences within the population’s preferences for primary care and groups or classes with homogeneous preferences are observable.

Considering the limited resources, health policy has to set priorities when attempting to meet these heterogeneous preferences for primary care provision. It is virtually not feasible to meet all specific needs. The attribute opening hours of the practice with the level 5 days a week had the largest effect on the respondents’ preferences in the conditional logit model and also in the two largest classes in the LCA. In terms of the future structuring of primary care this indicates that the rural population may not accept that care is provided only at limited times, e.g. in a branch of a primary care practice which opens 2–3 days a week. Other studies also show that a practice’s opening hours as well as the waiting time to an appointment are important aspects of primary care [24–26]. However, physician shortages in remote areas will probably not allow more rural practices with opening hours of 5 days a week. But this may not be absolutely necessary for patients, as the analysis of trade-offs between the attributes (see Table 4) showed that patients are willing to trade longer opening hours against a longer distance to the practice. Consequently, patients would be willing to accept a longer travelling time, e.g. to more urban areas, if a practice has extended opening hours. Extended opening hours can especially be implemented in group practices or medical care centers with two or more physicians. Besides longer opening hours, group practices may also offer extensive diagnostic facilities. This is also a significant attribute of care provision to the respondents, even though not as relevant as opening hours. Also for physicians a group practice seems to be an attractive workplace, as the number of GPs working in a group practice increased over the last years in Germany [27]. These arguments suggest a more centered primary care in rural areas.

A further important aspect of primary care, which has a significant effect in all 4 classes of the LCA, is the provision of home visits. They play an important role in the German healthcare system and are key tasks of primary care physicians [6, 28], particularly for the vulnerable group of old, multi-morbid and immobile persons who have specific needs concerning care provision. As rural and remote areas often have higher proportions of elderly residents and, at the same time, worse public transportation than in bigger cities, it is essential to maintain home visits in those regions. For those patients with particular need for home visits, the delegation of medical tasks may play a key role in the future primary care provision in rural areas, as nurse practitioners are able to relieve physicians with selected medical tasks and may, at the same time, even be able to take more time for the patients than physicians can.

While the attribute “delegation of medical tasks” does not have a significant effect on utility in the overall group of respondents, there is one latent class that prefers the delegation of medical tasks over care provision only by a physician. The non-significance of this attribute could mean that this attribute was not relevant for the respondents when choosing between the different choice alternatives or that there is a too large heterogeneity in the preferences of the overall group of in the respondents [28]. The latter reason is supported by the fact that this attributes has a significantly positive effect in class 2 and significantly negative effect in class 4.

Delegation of medical tasks is not preferred over care provision through a GP by most of the respondents. But even if patients do not specifically prefer delegation, other studies show that patients are willingness to use such a new model of care, if medical care in rural regions changes [29]. Further research and especially evaluation of existing (pilot) projects of the implementation of new models of care is needed to generate deeper insights into patients’ attitudes and preferences towards innovative care models.

### Limitations

This preference study has some limitations. In general, only a limited number of attributes and corresponding levels can be included in a DCE. Otherwise, the high complexity of the decision making would lead to a high cognitive burden for the respondents and the efficiency of the survey and the quality of the data obtained would decrease [13]. The selection of attributes and attribute levels is a central issue within a DCE. Although the selection of attributes and levels is based on a systematic literature search as well as on qualitative research – as it is recommended in various guidelines on DCE and Conjoint Analysis [7, 30] – it is possible that there are relevant attributes that were not included in the study. Furthermore, it is not possible to assess specific preferences for diverse new healthcare models, such as telemedicine or concepts based on mobility, in a DCE because these models of care are characterized by different attributes which cannot all be combined in one DCE. However, there are survey studies using e.g. rating scales published that report on the acceptance of new models of care [29].

A further limitation could be that due to the exclusion of an opt-out or status quo option which forced to respondents to make an explicit choice between the two alternatives. This may have led to missing data. In the literature, the inclusion of an opt-out potion is recommended if preferences for a good that is not consumed with certainty [19] which is not the case for primary care. In what way the exclusion of an opt-option led to missing data can unfortunately not be examined.

Although the self-administered postal questionnaire is the most common type of DCE [31], it is linked to some limitations. There is, for instance, no interviewer who could assist the study participants in answering the complex and cognitively demanding choice experiment. Therefore, we tried to keep the choice sets as simple as possible by using symbols for the attributes and levels and by using colors to mark the two choice alternatives.

Although the study faces some limitations, it provides valuable insights on the rural population’s preferences for various aspects of primary care.

## Conclusions

This study aimed at assessing the rural population’s preferences for primary healthcare in Germany. To this end, a DCE was conducted to assess preferences for a range of characteristics of primary care provision. The analysis revealed heterogeneous preferences among the study participants with some similarities. For example, opening hours of a practice and home visits are relevant aspects of healthcare for the majority of respondents.

During the last years primary care provision in Germany had to face some challenges. On the one hand, the demographic change led to an aging population which has increasing need for primary care. On the other hand, especially rural and remote regions face physician shortages. To react to this negative trend, current patient preferences can be used as a basis for restructuring care provision, e.g. in terms of promoting the implementation of more group practices or medical care centers or maintaining home visits. Nevertheless, it will not be possible for policy makers to address all needs.

If this trend continues, healthcare provision might in some regions only be maintained through the use of innovative models of care, such as delegation concepts, concepts based on mobility or telemedicine. In this case, it is important to continuously involve patients and assess their preferences also for specific new care models. Currently, the German population has varying acceptance of such new and innovative care concepts [26, 32]. To increase the acceptance, the population and especially patients should prospectively be more deeply involved in the organization and structuring of primary care provision.

## Additional file

Additional file 1: English version of the questionnaire and DCE. (PDF 841 kb)

## Data Availability

The datasets generated and analyzed during the current study are not publicly available. The dataset is housed at Hanover Medical School.

## Abbreviations

AIC: Akaike Information Criterion
BIC: Bayesian Information Criterion
DCE: Discrete choice experiment
GP: General practitioner
LCM: Latent class model
MRS: Marginal rate of substitution
No.: Number
Ref: Reference category
RUT: Random utility theory
SD: Standard deviation
SE: Standard error

## Footnotes

1 For more detailed information on the experimental design and construction of choice tasks see for example Amaya-Amaya [11].

2 Hard to serve regions are regions in which the need of medical care can no longer be met by traditional care models [3]. For a more detailed description of hard to serve regions see Tangermann et al. [14].
