# Supplementary material for: Public preferences for primary care provision in Germany – a discrete choice experiment
Source: BMC Fam Pract. 2019 Jun 11;20:80. doi: 10.1186/s12875-019-0967-y (PMC6560870; doi:10.1186/s12875-019-0967-y)
Supplement: Supplementary file 1 — English version of the questionnaire and DCE. (PDF 841 kb) [file 12875_2019_967_MOESM1_ESM.pdf]

Medizinische Hochschule Hannover

CHERH

Ein Projekt gefördert vom
Bundesministerium für Bildung und Forschung

# How do you evaluate primary care provision in your region?

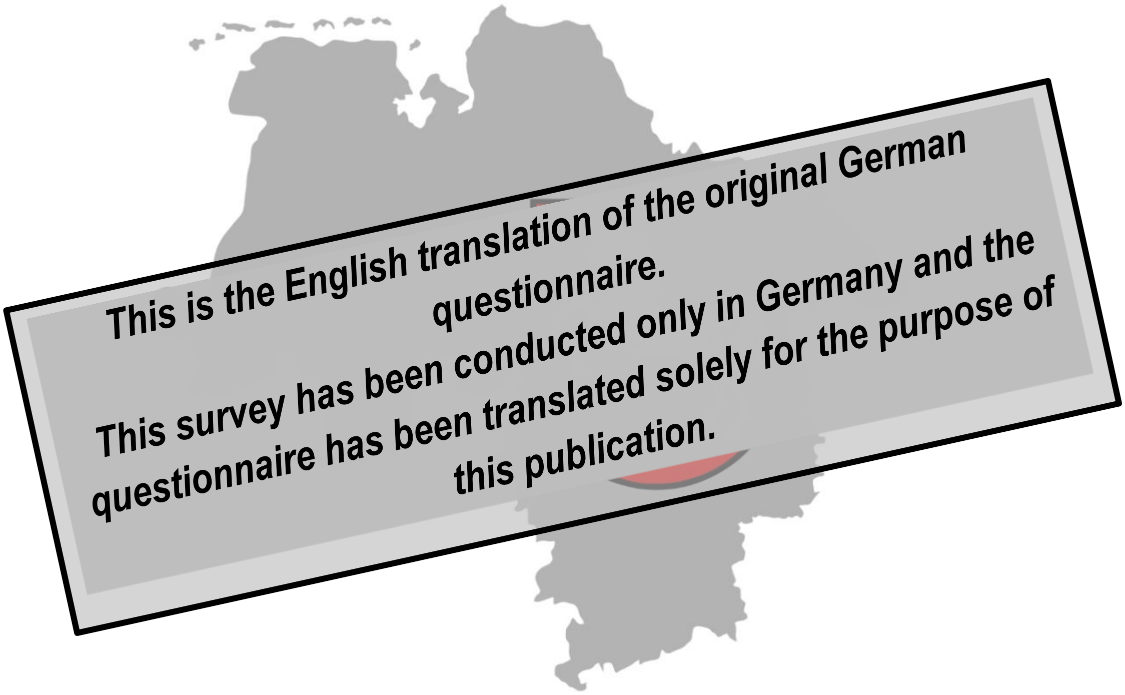


A survey conducted by Hannover Medical School
in 2015

Medizinische Hochschule Hannover

Dear participant,

The Hannover Medical School conducts a survey among the inhabitants of Lower Saxony's rural regions. This questionnaire includes questions on the current primary care provision as well as future development of primary care on-site.

It is our intention to improve primary care provision in Lower Saxony's rural regions und to take the population's needs and preferences into account. Therefore, we would like to determine how the population of rural regions assesses primary care and which aspects are of special importance for them.

The survey completion will take about 15 minutes.

**Information on the completion of the survey**

If you wish to correct an answer, please make the correction clearly visible and circle the correct answer: ■ ⊠

Please answer the questions in the right order, question by question. Do not skip any question except there is a clear notice to do so.

Please use a pen when completing the questionnaire.

In case you want to comment questions or give more detailed answers on some questions please use the last page of the questionnaire.

If you have any questions or need help completing the survey, please contact:


Contact person: [redacted]

Phone: [redacted]

Email: [redacted]


**Thank you for your participation!**

## PART I – PRIMARY CARE AND HEALTH STATUS

(1) Public media as well as policy currently discuss on primary care in rural and remote regions.

How do you personally rate the primary care provision at your site? Would you say the primary care provision is…

| Very good | Good | Moderate | Poor | Very poor |
| --- | --- | --- | --- | --- |
| ❑ | ❑ | ❑ | ❑ | ❑ |

(2) For how long have you already been patient of you current general practitioner?

- I don't have a general practitioner .................... ❑ ➡ ***Skip to question (6)***
- For less than 1 year .................... ❑
- For 1 – 5 years .................... ❑
- For 6 – 10 years .................... ❑
- For more than 10 years .................... ❑

(3) How long do you usually need to reach your general practitioner's practice form your home?
*(☞Please state the number of minutes – irrespectively of the transport you use to reach your general practitioner.)*

About __________ minutes

(4) How long do you usually wait for an appointment when visiting you general practitioner, if you have acute health complaints e.g. a flu?

- No appointment necessary .................... ❑
- No waiting time .................... ❑
- 1 day .................... ❑
- 2 days .................... ❑
- 3 days .................... ❑
- More than 3 days .................... ❑



MHH Medizinische Hochschule Hannover

(5) How often have you visited your general practitioner in the past 12 month for a treatment?

*(☞ Please count solely visits where you personally talked to the general practitioner.)*

Not at all ❑
1 – 3 times ❑
4 – 6 times ❑
7 – 10 times ❑
More than 10 times ❑

(6) Do you have the possibility to choose between different general practitioners at your site?

Yes ❑
No ❑

(7) How **important** are the following aspects of primary care for you personally?

| | Very unimportant | Rather unimportant | Partly important | Rather important | Very important |
|---|---|---|---|---|---|
| Short waiting time for an appointment | ❑ | ❑ | ❑ | ❑ | ❑ |
| Short distance to the practice | ❑ | ❑ | ❑ | ❑ | ❑ |
| Personal relationship to the general practitioner | ❑ | ❑ | ❑ | ❑ | ❑ |
| Extensive diagnostic facilities of the practice | ❑ | ❑ | ❑ | ❑ | ❑ |
| Professional skills of the general practitioner | ❑ | ❑ | ❑ | ❑ | ❑ |
| Provision of home visits | ❑ | ❑ | ❑ | ❑ | ❑ |

(8) How **satisfied** are you with the following aspects of primary care at your site?

| | Very unsatisfied | Rather unsatisfied | Partly (un)satisfied | Rather satisfied | Very satisfied | I don't know |
|---|---|---|---|---|---|---|
| Waiting time for an appointment | ❑ | ❑ | ❑ | ❑ | ❑ | ❑ |
| Distance to the practice | ❑ | ❑ | ❑ | ❑ | ❑ | ❑ |
| Relationship to the general practitioner | ❑ | ❑ | ❑ | ❑ | ❑ | ❑ |
| Diagnostic facilities of the practice | ❑ | ❑ | ❑ | ❑ | ❑ | ❑ |
| Professional skills of the general practitioner | ❑ | ❑ | ❑ | ❑ | ❑ | ❑ |
| Provision of home visits | ❑ | ❑ | ❑ | ❑ | ❑ | ❑ |

(9) Could you imagine moving, in case the primary care provision is no longer sufficient?

Yes, for sure ........................................ ❑

It depends ........................................ ❑

No, nothing doing ........................................ ❑

(10) Do you think that primary care provision at your site will change within the next 5 years? Would you say it will…

…improve ........................................ ❑

…stay the same ........................................ ❑

…worsen ........................................ ❑

(11) Do you think the following aspects of primary care at your site will **change** within the next 5 years?

| | Strongly worsen | Slightly worsen | Stay the same | Slightly improve | Strongly improve | I don't know |
|---|---|---|---|---|---|---|
| Waiting time for an appointment | ❑ | ❑ | ❑ | ❑ | ❑ | ❑ |
| Distance to the practice | ❑ | ❑ | ❑ | ❑ | ❑ | ❑ |
| Relationship to the general practitioner | ❑ | ❑ | ❑ | ❑ | ❑ | ❑ |
| Diagnostic facilities of the practice | ❑ | ❑ | ❑ | ❑ | ❑ | ❑ |
| Professional skills of the general practitioner | ❑ | ❑ | ❑ | ❑ | ❑ | ❑ |
| Provision of home visits | ❑ | ❑ | ❑ | ❑ | ❑ | ❑ |

The next questions will focus on your personal health status.

(12) How would you describe your current general health?

| Excellent | Very good | Good | Less good | Poor |
|---|---|---|---|---|
| ❑ | ❑ | ❑ | ❑ | ❑ |

(13) Do you suffer from a chronic disease (e.g. diabetes or asthma)?

Yes ........................................................................................................................ ❑

No .......................................................................................................................... ❑ ➡ ***Continue on page 5***

(14) Are you restricted in your daily life due to this disease?

Yes, I am very restricted .................................................................................... ❑

Yes, I am somewhat restricted ........................................................................... ❑

No, I am not restricted ....................................................................................... ❑

## Part II – Options of Primary Care Provision

On the next page you will see a selection of fictional primary care situations. 9 different choice situations will compare two situations where the following attributes (home visits, distance to practice, healthcare provisions by… etc.) are continuously changing. Please choose each time the one situation you prefer over the other one. You cannot give any “right” or “wrong” answers. We are only interested in your personal opinion.

The following six attributes of primary care will appear repeatedly on the following pages of the questionnaire:

### Description of the attributes of primary care

**Home visits:** Medical care at the patients‘ home in case they are not able to get to the practice.

**Distance to the practice:** Number of minutes that patients need to reach the general practitioner’s practice (irrespectively of the transport they use to reach the practice).

**Healthcare provision by…:** Healthcare on-site through one physician / several varying physicians.

**Opening hours of the practice:** Days on which the practice opens and a physician is present.

**Delegation of medical tasks:** Delegation of simple tasks to professionals who have specific qualification (e. g. administration of pharmaceuticals if the physician prescribed it, care of wounds or vaccinations).

**Diagnostic facilities of the practice:** Medical-laboratory facilities for diagnostics and treatment of diseases at the family practice (e. g. via ultrasound, ECG, pulmonary function test). Limited diagnostic facilities might restrict possibilities for diagnostics and medical care and lead to referrals to specialists.



MHH Medizinische Hochschule Hannover

On the following pages a total of 9 choice sets will be presented which show different options of primary care provision. Please choose the one option you prefer over the other one in each of the choice sets.
Imagine that the current primary care provision on-site does not exist any longer but instead one of the primary care situations as described below would be comprised.

1. Which of the two primary care situations (situation 1 *or* situation 2) would you prefer? Please tick your preferred situation:

| Attribute | Situation 1 | Situation 2 |
|---|---|---|
| Home visits | Home visits are provided | Home visits are <u>not</u> provided |
| Distance to the practice | 15 minutes | 45 minutes |
| Healthcare provision by… | … <u>one</u> physician | … <u>several varying</u> physicians |
| Opening hours of the practice | Practice opens <u>five days</u> a week | Practice opens <u>three days</u> a week |
| Delegation of medical tasks | No delegation of medical tasks to specially trained professionals | Delegation of medical tasks to specially trained professionals |
| Diagnostic facilities of the practice | Extensive diagnostic facilities | Limited diagnostic facilities |
| **I choose…** | **situation 1** ☐ | **situation 2** ☐ |

2. Which of the two primary care situations (situation 1 *or* situation 2) would you prefer? Please tick your preferred situation:

| Attribute | Situation 1 | Situation 2 |
|---|---|---|
| Home visits | Home visits are provided | Home visits are <u>not</u> provided |
| Distance to the practice | 15 minutes | 30 minutes |
| Healthcare provision by… | … <u>one</u> physician | … <u>several varying</u> physicians |
| Opening hours of the practice | Practice opens <u>three days</u> a week | Practice opens <u>four days</u> a week |
| Delegation of medical tasks | No delegation of medical tasks to specially trained professionals | Delegation of medical tasks to specially trained professionals |
| Diagnostic facilities of the practice | Extensive diagnostic facilities | Limited diagnostic facilities |
| **I choose…** | **situation 1** ☐ | **situation 2** ☐ |

**3.** Which of the two primary care situations (situation 1 *or* situation 2) would you prefer? Please tick your preferred situation:

| **Attribute** | **Situation 1** | **Situation 2** |
|---|---|---|
| Home visits | Home visits are provided | Home visits are not provided |
| Distance to the practice | 15 minutes | 45 minutes |
| Healthcare provision by… | … one physician | … several varying physicians |
| Opening hours of the practice | Practice opens four days a week | Practice opens three days a week |
| Delegation of medical tasks | Delegation of medical tasks to specially trained professionals | No delegation of medical tasks to specially trained professionals |
| Diagnostic facilities of the practice | Extensive diagnostic facilities | Limited diagnostic facilities |
| **I choose…** | **situation 1** ☐ | **situation 2** ☐ |

**4.** Which of the two primary care situations (situation 1 *or* situation 2) would you prefer? Please tick your preferred situation:

| **Attribute** | **Situation 1** | **Situation 2** |
|---|---|---|
| Home visits | Home visits are provided | Home visits are not provided |
| Distance to the practice | 30 minutes | 15 minutes |
| Healthcare provision by… | … one physician | … several varying physicians |
| Opening hours of the practice | Practice opens five days a week | Practice opens four days a week |
| Delegation of medical tasks | Delegation of medical tasks to specially trained professionals | No delegation of medical tasks to specially trained professionals |
| Diagnostic facilities of the practice | Extensive diagnostic facilities | Limited diagnostic facilities |
| **I choose…** | **situation 1** ☐ | **situation 2** ☐ |

**5.** Which of the two primary care situations (situation 1 *or* situation 2) would you prefer? Please tick your preferred situation:

| **Attribute** | **Situation 1** | **Situation 2** |
|---|---|---|
| Home visits | Home visits are provided | Home visits are <u>not</u> provided |
| Distance to the practice | 15 minutes | 30 minutes |
| Healthcare provision by… | … <u>several varying</u> physicians | … <u>one</u> physician |
| Opening hours of the practice | Practice opens <u>three days</u> a week | Practice opens <u>four days</u> a week |
| Delegation of medical tasks | Delegation of medical tasks to specially trained professionals | No delegation of medical tasks to specially trained professionals |
| Diagnostic facilities of the practice | Limited diagnostic facilities | Extensive diagnostic facilities |
| **I choose…** | **situation 1** ☐ | **situation 2** ☐ |

**6.** Which of the two primary care situations (situation 1 *or* situation 2) would you prefer? Please tick your preferred situation:

| **Attribute** | **Situation 1** | **Situation 2** |
|---|---|---|
| Home visits | Home visits are <u>not</u> provided | Home visits are provided |
| Distance to the practice | 15 minutes | 45 minutes |
| Healthcare provision by… | … <u>one</u> physician | … <u>several varying</u> physicians |
| Opening hours of the practice | Practice opens <u>three days</u> a week | Practice opens <u>four days</u> a week |
| Delegation of medical tasks | Delegation of medical tasks to specially trained professionals | No delegation of medical tasks to specially trained professionals |
| Diagnostic facilities of the practice | Extensive diagnostic facilities | Limited diagnostic facilities |
| **I choose…** | **situation 1** ☐ | **situation 2** ☐ |

7. Which of the two primary care situations (situation 1 *or* situation 2) would you prefer? Please tick your preferred situation:

| Attribute | Situation 1 | Situation 2 |
| --- | --- | --- |
| Home visits | Home visits are not provided | Home visits are provided |
| Distance to the practice | 45 minutes | 30 minutes |
| Healthcare provision by… | … one physician | … several varying physicians |
| Opening hours of the practice | Practice opens four days a week | Practice opens five days a week |
| Delegation of medical tasks | Delegation of medical tasks to specially trained professionals | No delegation of medical tasks to specially trained professionals |
| Diagnostic facilities of the practice | Extensive diagnostic facilities | Limited diagnostic facilities |
| **I choose…** | situation 1 ☐ | situation 2 ☐ |

8. Which of the two primary care situations (situation 1 *or* situation 2) would you prefer? Please tick your preferred situation:

| Attribute | Situation 1 | Situation 2 |
| --- | --- | --- |
| Home visits | Home visits are provided | Home visits are not provided |
| Distance to the practice | 30 minutes | 45 minutes |
| Healthcare provision by… | … one physician | … several varying physicians |
| Opening hours of the practice | Practice opens five days a week | Practice opens three days a week |
| Delegation of medical tasks | Delegation of medical tasks to specially trained professionals | No delegation of medical tasks to specially trained professionals |
| Diagnostic facilities of the practice | Limited diagnostic facilities | Extensive diagnostic facilities |
| **I choose…** | situation 1 ☐ | situation 2 ☐ |

**9.** Which of the two primary care situations (situation 1 *or* situation 2) would you prefer? Please tick your preferred situation:

| **Attribute** | **Situation 1** | **Situation 2** |
|---|---|---|
| Home visits | Home visits are not provided | Home visits are provided |
| Distance to the practice | 45 minutes | 15 minutes |
| Healthcare provision by… | … one physician | … several varying physicians |
| Opening hours of the practice | Practice opens five days a week | Practice opens four days a week |
| Delegation of medical tasks | No delegation of medical tasks to specially trained professionals | Delegation of medical tasks to specially trained professionals |
| Diagnostic facilities of the practice | Extensive diagnostic facilities | Limited diagnostic facilities |
| **I choose…** | situation 1 ☐ | situation 2 ☐ |

(15) If you think about the nine choices you just had to make, what would you say: How difficult has it been to choose one of the two situations?

| Not difficult | Rather not difficult | Rather difficult | Difficult |
|---|---|---|---|
| ❑ | ❑ | ❑ | ❑ |

(16) How sure have you been in choosing a situation?

| Not sure | Rather not sure | Rather sure | Sure |
|---|---|---|---|
| ❑ | ❑ | ❑ | ❑ |

## PART III – PERSONAL INFORMATION

(17) Your gender?

Female ........................................................................................................................ ❑

Male ............................................................................................................................ ❑

(18) In which year have you been born?

| 1 | 9 | | |
|---|---|---|---|

(19) How many patients do permanently live in your household, including yourself?

A total of ____________ person(s)

Thereof __________ child(ren) under the age of 18 years

(20) What is your current employment status?

Full-time employed .......................................................................................... ❑

Part-time employed ......................................................................................... ❑

Marginally or irregularly employed ..................................................................... ❑

Company-based training / company-based retraining ....................................................... ❑

School / University ............................................................................................ ❑

Voluntary military service ..................................................................................... ❑

Voluntary social/ecological year, “Bundesfreiwilligendienst ............................................... ❑

Retired ................................................................................................................. ❑

Currently not employed ........................................................................................ ❑

Other: ____________________________________________

(21) What is you highest level of school-leaving qualification?

Still going to school ........................................................................................ ❑

No school-leaving qualification ........................................................................ ❑

School-leaving qualification “Volks- oder Hauptschulabschluss“ ..................................... ❑

School-leaving qualification “Realschulabschluss / Mittlere Reife“ ..................................... ❑

School-leaving qualification “Fachhochschulreife“ ............................................................ ❑

School-leaving qualification “Abitur / Hochschulreife”......................................................... ❑

Other school-leaving qualification: ____________________________

(22) Which kind of health insurance are you insured at?

*(☞Please answer this question even if you are not insured independently but as a family member.)*

Insured in a statutory health insurance ................................................................. ❑

Solely insured in a private health insurance ....................................................... ❑

If you should have questions or comments on this survey, please feel free to make notes here:

_______________________________________________

_______________________________________________

_______________________________________________

_______________________________________________

_______________________________________________

_______________________________________________

_______________________________________________

**Thank you for your participation!**
